# Preparation of ethyl levulinate from wheat stalk over Zr(SO_4_)_2_/SiO_2_

**DOI:** 10.3906/kim-2010-67

**Published:** 2021-08-27

**Authors:** Ding-kai WANG, Wei ZHAO, Ming-yu CUI, Tian-tian GUO, Shui-yuan FU, Wei-gang LI

**Affiliations:** 1 Key Laboratory of Coal Processing and Efficient Utilization, China University of Mining & Technology, Jiangsu China

**Keywords:** Ethyl levulinate, glucose, solid acid Zr(SO_4_)_2_/SiO_2_, wheat stalk

## Abstract

A series of Zr(SO_4_)_2_/SiO_2_ solid acid catalysts with different Zr(SO_4_)_2 _loadings were prepared by water-soluble-impregnation method at room temperature. Then, the prepared catalysts were characterized by Fourier transform infrared spectroscopy, transmission electron microscopy and energy-dispersive X-ray spectrum, X-ray diffraction, adsorption/desorption of N_2_, and temperature-programmed desorption of NH_3_. The results showed that the active component Zr(SO_4_)_2_ was successfully adhered to the mesoporous SiO_2_, and the acid amount of Zr(SO_4_)_2_/SiO_2_ increased with the increasing of the Zr(SO_4_)_2_ loadings. Finally, the wheat stalk was used as raw material and depolymerized over Zr(SO_4_)_2_/SiO_2 _to produce ethyl levulinate (EL). The reaction mixture was separated and purified by filtration and vacuum distillation. The kinetic characteristics and the reaction pathway were also studied. A comparative study showed that 20 wt.% Zr(SO_4_)_2_/SiO_2_ exhibited higher catalytic activity. When reaction temperature, time, catalyst dosage and Zr(SO_4_)_2_ loadings were 190 °C, 50 min, 20 wt.% and 30 wt.%, the EL yield reached a maximum of 17.14%. The relative content of EL exceeded 90% after three steps of distillation.

## 1. Introduction

With the development of economy, fossil resources, such as coal, oil and natural gas, are almost exhausted, and serious environmental problems have been caused. It is extremely urgent to make full use of green and renewable resources [1–3]. Biomass resource is the only renewable carbon source that can be used as the raw material for the productions of carbon-based chemicals and energy [4]. As an important biomass, crop straw with its great abundance and high utilization potential has attracted more and more attention [5–9]. The use of crop straw as a raw material for the production of value-added chemicals can obtain great economic benefit. As a kind of crop straw, wheat stalk is mainly composed of cellulose, hemicellulose, and lignin. Among them, cellulose, which accounts for 45% of wheat straw, is the main substrate for catalytic conversion to biofuel [10–12]. In previous studies on the preparation of valuable platform compounds, a relatively simple and effective way is the catalytic conversion of biomass-derived sugars to ethyl levulinate (EL) using various catalysts [13]. However, less attention has been paid to the conversion of crop straw to EL using an efficient catalyst. Based on current research status of crop straw utilization, it is important to increase the yield of valuable chemicals from direct conversion of crop straw [14–16].

As a chemical product with wide industrial application prospect, EL has been used in the fields of petroleum additives, perfume and pharmaceutical intermediates [17,18]. In addition, EL, being one of the levulinate esters (LE), contains ~14 mol% oxygen and has similar properties with fatty acid ethyl esters in biodiesel [19]. EL is added to diesel oil to form a kind of biodiesel fuel, which has high lubricity, flash point stability, low sulfur content, and suitable viscosity, and can be used in conventional diesel engine [20]. These promising market potentials urge the development of technologies on improving the efficiencies of producing EL, especially through cost-effective and environmentally friendly methods. To date, several researchers have reported the conversion of sucrose, cellulose, and biomass wastes into LE using inorganic liquid acids (especially sulfuric acid) as catalyst. For instance, Mascal et al. [21] commented on the processes for one-pot conversion of cellulose into EL, and the EL yield is on the order of 20%. Mao et al. [22] reported one pot two-step synthesis process for producing EL from paper pulp over H_2_SO_4_, and the EL yield reached 25.9 wt.%. Although these reactions were effective, the inorganic liquid acids have serious drawbacks in the aspects of separation and recycling, as well as equipment corrosion [23]. So, it is extremely important and necessary to develop new and environmentally benign catalysts with high activity for the production of EL. In recent decades, solid acid catalyst as a heterogeneous catalyst has attracted extensive interest. It can overcome the above disadvantages of the inorganic acid in acid catalysis and has been widely applied to catalyze dehydration, alkylation, cracking, isomerization, esterification, acylation, and so on [24–27]. Among various solid acid catalysts, sulfated metal oxides have been widely concerned by researchers because of their strong acidity and excellent thermal stability. Therefore, sulfated metal oxides are expected to show high catalytic activity for the conversion of biomass to LE. For example, Sun et al. [28] applied a solid acid catalyst, USY, to convert cellulose to EL under anhydrous conditions, and the yield of ethyl levulinate reached 14.95%, whilst Li et al. [29] converted cellulose to EL over the composite solid acid S_2_O_8_^2–^/ZrO_2_/USY and the yield of EL reached 34.6%. Chang et al. [30] used SO_4_^2–^/ZrO_2_/USY to catalyze the alcoholysis of cellulose to prepare EL, and the yield of EL was also significantly improved. In the early days, the researchers found that Zr(SO_4_)_2_∙4H_2_O has a highly acidity and a layered structure [31]. Zr^4+^ is in a state of severe electron deficiency and forms a strong coordination bond with oxygen atoms in bound water. On the other hand, oxygen atoms in sulfate radical form hydrogen bonds with bound water. Under the influence of these two aspects, the hydrogen in the water is severely delocalized, thus forming a Brønsted acid center [32,33]. Since Zr(SO_4_)_2_ has a small specific surface area, it is supported on a carrier with a high specific surface and dispersed on the carrier to expose acid active sites [34]. Mesoporous SiO_2_ is a carrier with high specific surface area and loading Zr(SO_4_)_2_ onto the carrier will maximize its catalytic activity [35–37].

In this work, the highly acidic Zr(SO_4_)_2_ was loaded on mesoporous SiO_2_ with high specific surface area (SSA) to prepare Zr(SO_4_)_2_/SiO_2_ catalysts, which could solve the drawbacks of inorganic acid catalysts, improve the acidity of the catalysts, and then increase the catalytic activity. Then, wheat stalk powder (WSP) with complex structure was used as the raw material to produce EL over Zr(SO_4_)_2_/SiO_2_ catalyst in an ethanol-cyclohexane system. The liquid product obtained from depolymerization of WSP was effectively separated by rotary evaporation. These results will be helpful to get insight into the conversion process of biomass to EL, and produce EL from wheat stalk by one-pot.

## 2. Materials and methods

### 2.1. Materials

Wheat stalk was collected from Xuzhou, Jiangsu, China. They were washed with water and then dried in sunlight, chopped into small pieces, and pulverized to pass through an 80-mesh sieve (<180 µm) to obtain wheat stalk powder (WSP). Tetraethyl orthosilicate (TEOS, 99%), ammonia solution, cetyltrimethylammonium bromide (CTAB, 99%), ethyl alcohol, and zirconium sulfatetetrahydrate were purchased from Sinopharm Chemical Reagent Co., Ltd. Cyclohexane was purchased from Aladdin Reagent Co., Ltd.

### 2.2. Preparation and characterization of catalyst 

Mesoporous silica was prepared based on the method found in the literature [38]. Typically, CTAB (1 g) was completely dissolved in aqueous ammonia (1.2 mol/L, 110 mL) by ultrasonic vibration. Then, TEOS (5 mL) was added into the above solution. After stirring for 24 h at room temperature, the mixture was filtered and washed with deionized water to obtain a white solid, which was immersed in deionized water (400 mL) for 24 h. Then it was filtered and dried at 80 °C for 6 h. Finally, the mesoporous silica was obtained through calcining at 500 °C for 6 h, and subsequently employed as a support for the preparation of the solid acid catalyst.

Zr(SO_4_)_2_/SiO_2_ solid acid catalyst was synthesized by the Stöber process [39]. A certain amount of Zr(SO_4_)_2_ was dissolved in deionized water (15 mL) by ultrasonic vibration, followed by adding the support SiO_2_ (0.4 g) under magnetic stirring and being immersed at room temperature for 24 h. Then the water was evaporated and the left mixture was dried in a vacuum oven at 80 °C for 6 h to obtain Zr(SO_4_)_2_/SiO_2_ solid acid catalyst.

The Fourier transform infrared (FTIR) spectra of the catalysts were recorded on a Nicolet Magna IR-560 FTIR spectrometer from 400 to 4000 cm^–1^. Scanning electron microscopy (SEM) investigations were made using Quanta 200 (FEI, USA). Transmission electron microscopy (TEM) images were obtained from Tecnai-G2-F20 TEM (FEI, USA) with 0.14 nm of resolution combined with an energy dispersive spectrometer. X-ray diffraction (XRD) patterns were recorded with a Bruker D8 ADVNCE X-ray diffractometer (Bruker, Germany). The X-ray tube uses Cu as target and released Kα radiation when accelerated at 30 mA and 40 kV. The scanning rate and 2θ scanning angle range are 0.19451^o^ per s and 3° to 90°, respectively. SSA, total pore volume (TPV) and average pore diameter (APD) of the catalysts were measured by nitrogen physical adsorption, using an autosorb-1 (Quantachrome, USA) at –196 °C. Before the tests, each sample was evacuated at 120 °C for 10 h.

Temperature-programed desorption of ammonia (NH_3_-TPD), which was used to determine acid strength of catalysts, was carried out by a Quantachrome automated chemisorption flow analyzer with a TCD detector. Before each test, 30 mg sample was placed in a U-type quartz tube, flushed by helium flow at 120 °C for 60 min and cooled to 50 °C. After the saturated adsorption of NH_3_ on the catalyst surface, the sample was kept at 50 °C in helium flow for 60 min to remove physically adsorbed NH_3_. The desorption of NH_3_ was carried out from 50 °C to 600 °C at a heating rate of 15 °C·min^–1^.

### 2.3. Preparation of EL from WSP

WSP (2.5 g), anhydrous ethanol (34 mL), cyclohexane (17 mL) and a certain amount of catalyst Zr(SO_4_)_2_/SiO_2_ were added into a 200 mL stainless steel autoclave. After purging the air out of the autoclave with a vacuum pump at room temperature, the autoclave was heated to a certain temperature (160–220 °C) within 15 min and kept at that temperature for 30–70 min (reaction pressure varies with reaction temperature and the amounts of WSP and solvents). After the reaction was completed, the mixture was taken out from the autoclave and filtrated through a 0.8 µm membrane to obtain EL solution. Each experiment under the same conditions was repeated at least 3 times and the errors of the yields of EL based on WSP and the residue yields are within ±1%.

###  2.4. Separation of EL. 

**Figure 1:**
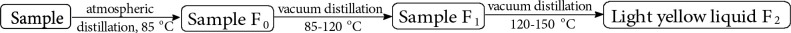
Flowchart of EL separation.

A three-step distillation method was used to separate EL from the obtained EL solution. The detailed process was shown in Figure 1. Firstly, the sample was distilled at 85 °C under normal pressure and the remainder was recorded as F_0_. Then, sample F_0_ was distilled under reduced pressure during 85–120 °C and the remainder was recorded as F_1_. Sample F_1_ was then treated by vacuum distillation between 120 °C and 150 °C. The last residue is light yellow liquid and is denoted as F_2_. The composition of sample F_2_ was analyzed with a Hewlett-Packard 6890/5973 gas chromatography-mass spectrometry (GC-MS) and an Agilent 1260 high performance liquid chromatogram (HPLC).

###  2.5. Analysis and calculation methods. 

The concentrations of EL in the filtrate were determined by HPLC, equipped with a ZORBAX Eclipse Plus C_18_ column (4.6 × 250 mm, 5 m) and a diode array detector. The wavelength of chromatograph was set at 268 nm. In isocratic elution mode, a mixture of methanol and water (1:1 volume) was used as the mobile phase at a flow-rate of 1 mL.min^–1^. The concentration of EL (C_EL_) was determined via the external standard method, and the yield of EL based on WSP (Y_EL_) was calculated according to the following formulae:

Y_EL_ (%) = C_EL_V/1000M_1 _× 100% (based on WPS).

Where, C_EL_ is concentration of EL (mg mL^–1^), V is volume of the filtrate (mL), M_1_ is mass of WSP (g).

The residue yield (Y_R_, %) was calculated as the mass ratio of residue (M_3_−M_2_, M_3_, and M_2_ denote the mass of filter cake and catalyst, respectively) and WSP (M_1_) on dry basis, i.e., Y_R _= (M_3_−M_2_)/M_1 _× 100.

Y_EL_’ and Y_R_’ denote EL yield and residue yield when no catalyst is used.

### 2.6. Influence transport phenomena on the catalysts

Investigation of transport influence in heterogeneous catalysis is of vital importance especially in a system that involves transfer of bulky molecules. This is investigated by using turnover frequency (TOF) value to comparatively check the activity of the catalyst. TOF is defined as the moles reacted per s per surface mole of the active species [40]. It quantifies the activity of the active center for catalytic reaction under a specific reaction condition by the number of molecules converted per unit time [41].

Where m_EL _is the amount in moles of EL (mmol); t is the reaction time (min); f_m _is the amount of acid sites on the surface (mmol×g^–1^).

## 3. Results and discussion

3.1. Characterization of catalyst Zr(SO_4_)_2_/SiO_2_

**Figure 2:**
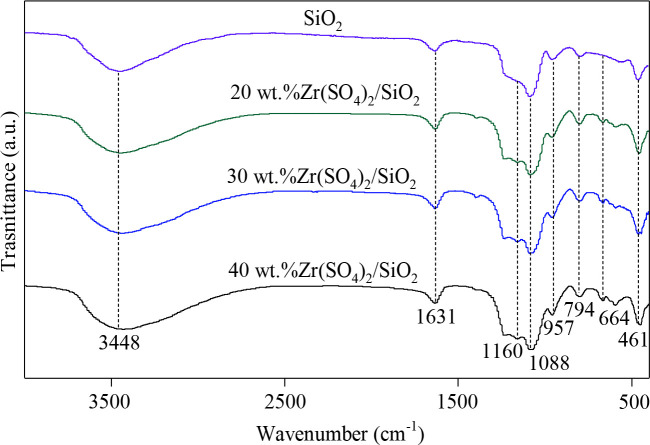
FTIR spectra of support and catalysts with different Zr(SO_4_)_2_ loadings.

FTIR spectra of the support and catalysts are shown in Figure 2. The infrared adsorption bands at 3448 cm^–1^ and 1631 cm^–1^ are attributed to the stretching frequency of physical adsorbed water [42], indicating that the sample contains a small amount of water. The peaks at 1088, 957, 794, and 461 cm^–1^ are assigned to the asymmetric stretching vibration of Si-O-Si, the stretching vibration of Si-OH, the symmetric stretching vibration of Si-O-Si and the bending vibration of Si-O-Si, respectively [43], indicating that the support may be SiO_2_. In addition, no absorption peak of methylene was detected in the support, indicating that CTAB was completely removed. The catalysts with different loadings of Zr(SO_4_)_2_ still have characteristic peaks of SiO_2_, indicating that SiO_2_ still exists on the catalysts. In addition, the FTIR spectra of catalysts with different Zr(SO_4_)_2_ loadings show the absorption peaks at 1160 and 664 cm^–1^, and the intensity of these peaks increases with the increasing of Zr(SO_4_)_2_ loading. The adsorption band at 1160 cm^–1^ is characteristic of the asymmetric stretching vibration of S=O and the peak at 664 cm^–1^ was attributed to the asymmetric flexural vibration of O=S=O [44]. The existence of these peaks proves that the active component Zr(SO_4_)_2_ is loaded on the support.

**Figure 3:**
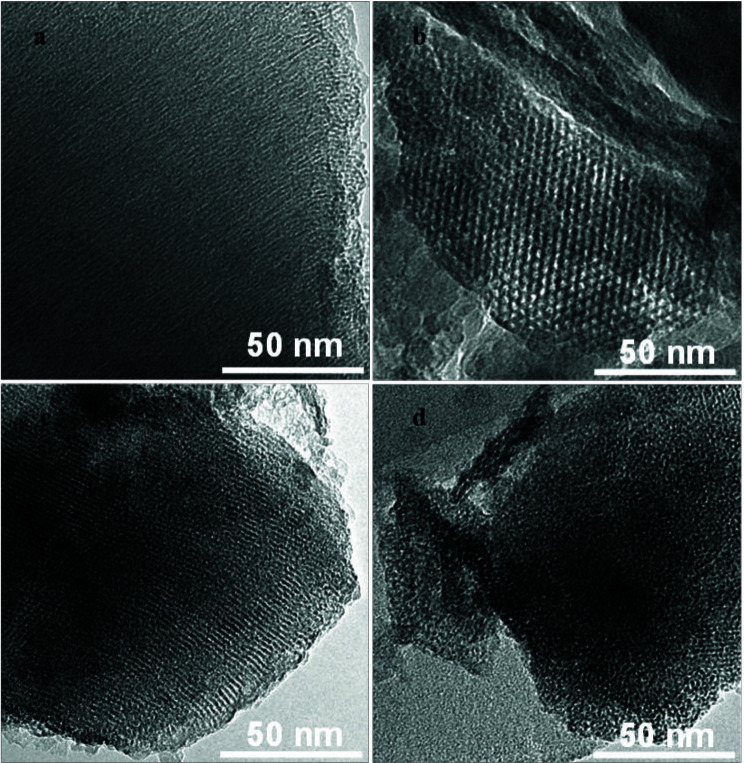
TEM images of samples: (a) SiO_2_ in top view; (b) SiO_2_ in side view; (c) Zr(SO_4_)_2_/SiO_2_ (30 wt.%) in top view; (d) and Zr(SO_4_)_2_/SiO_2_ (30 wt.%) in side view.

From Figures 3a and 3b, a honeycomb-like pore structure can be observed in the TEM image of the support, because that CTAB forms a one-dimensional hexagonal ordered structure under alkaline condition [45,46]. The support SiO_2_ was prepared through the aggregation of CTAB as structure-directing agent on the surface of the silica core under basic conditions, followed by subsequent hydrolysis and condensation of the TEOS as silica source [47]. When CTAB is decomposed during calcination, this regular pore structure can be formed. After Zr(SO_4_)_2_ (30 wt.%) is loaded, this pore structure becomes somewhat ambiguous as shown in Figures 3c and 3d. The possible reason is that the active component Zr(SO_4_)_2_ is filled into the pores of the support.

From EDS of the catalyst and support in Figure SI1 (left), the atom ratio (at, %) of Si and O is 1:2 in the support (as shown in Table 1). Furthermore, there are no other elements showed. During the test of TEM-EDS, C is caused by the carbon film as the bottom plate, while Cu is caused by the support of the copper mesh. All of these indicate that the prepared support is SiO_2_. Besides Si and O elements, Zr and S elements are also detected in the catalyst, indicating that the active component is loaded on the support. As shown in Table 1 and Table 2, the atomic percentage of O element increases from 66.60% to 67.36%, which also proves that the active component is loaded on the support.

**Table 1:** Relative element content of support.

Element	Wt (%)	At (%)
Si	46.70	33.30
O	53.30	66.60

**Table 2:** Relative element content of Zr(SO_4_)_2_/SiO_2_ (30 wt.%).

Element	Wt (%)	At (%)
Si	38.82	28.92
O	51.67	67.36
S	3.65	2.38
Zr	5.85	1.34

**Figure 4:**
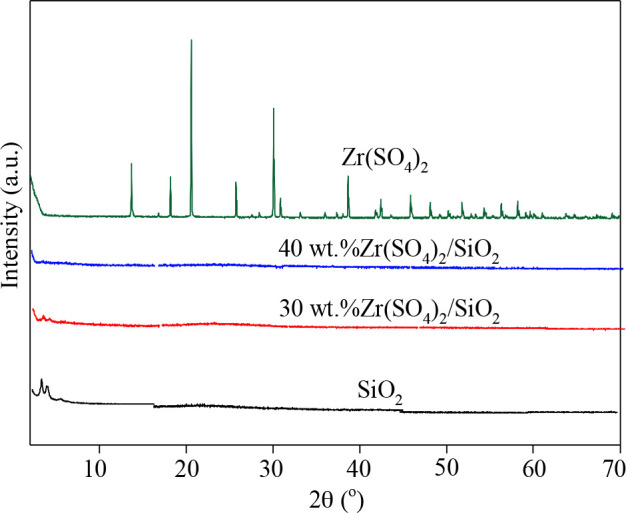
XRD patterns of SiO_2_ and Zr(SO_4_)_2_/SiO_2_ with different Zr(SO_4_)_2_ loadings.

As shown in Figure 4, there are no obvious characteristic diffraction peaks of SiO_2_ in XRD pattern of the support, indicating that the support SiO_2_ is amorphous. The strong diffraction peaks at 13.50, 18.01, 20.54, 25.60, 29.60, 29.00, 30.70, 38.60, 42.30, and 45.80° are the characteristic peaks of Zr(SO_4_)_2_ [48]. The characteristic peaks of Zr(SO_4_)_2 _don^’^t appear in the XRD patterns of catalysts with 30 wt.% and 40 wt.% Zr(SO_4_)_2_ loadings, but FTIR and TEM-EDS analysis show that the active component Zr(SO_4_)_2_ is loaded on the support, indicating that the active component Zr(SO_4_)_2_ is greatly dispersed on the support, which will also facilitate subsequent reactions.

**Figure 5:**
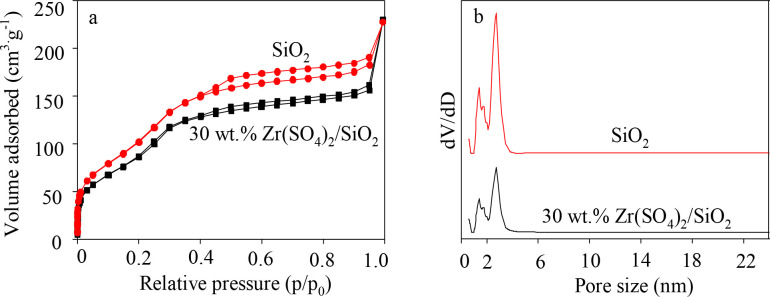
N_2_ adsorption/desorption isotherms (a) and pore size distribution (b) of support and catalyst (30 wt.% Zr(SO_4_)_2_/SiO_2_).

The N_2_ adsorption-desorption isotherms and pore size distribution of the prepared support and catalyst (30 wt.% Zr(SO_4_)_2_/SiO_2_) are depicted in Figure 5. As shown in Figure 5a, the N_2_ adsorption-desorption isotherms of support and catalyst are conventional IV type, and a typical H3 hysteresis loop appears in each curve, which proves that the sample contains a large number of micropores and mesopores [49]. From Figure 5b, the peak of the support at 2 nm is stronger, and there is also a weaker peak at less than 2 nm, indicating that the support is a mesoporous material with partial microporous. In the pore size distribution diagram of the catalyst, the peak shape and location of the catalyst are similar to those of the support, indicating that the loading process does not damage pore structure of the support. As listed in Table 3, the SSA falls down from 1166.11 in the support to 585.77 m^2^g^–1 ^in the catalyst after loading Zr(SO_4_)_2_ (30 wt.%), TPV decreases from 0.783 to 0.433 cm^3^g^–1^, and APD increases from 2.679 to 2.985 nm. All of these may be attributed to the load of Zr(SO_4_)_2_ on the carrier. The load of Zr(SO_4_)_2_ blocked a small fraction of the pores, which leaded to the decreasing of SSA and TPV. Furthermore, part of the pores was corroded due to the strong acidity of Zr(SO_4_)_2_, which also caused the increasing of APD. Table SI1 presents the results of the surface area and porosity analysis of the catalysts with various Zr(SO_4_)_2_/SiO_2_ loading except 30 wt.% Zr(SO_4_)_2_/SiO_2_. The result showed that SSA decreased gradually from 1166.11 to 479.73 m^2^∙g^–1^ with increasing Zr(SO_4_)_2_ loading, which was is because of the load of Zr(SO_4_)_2_ on the carrier.

**Table 3:** Surface properties of SiO2 and Zr(SO4)2

Sample	SSA (m2∙g–1)a	TPV(cm3∙g–1)b	APD (nm)b
SiO2	1166.11	0.783	2.687
Zr(SO4)2/SiO2	585.77	0.433	2.954

a SSA was calculated using the Brunauer–Emmett–Teller equation.b TPV and average pore diameter were estimated using the Barret–Joyner–Halenda model.

**Figure 6:**
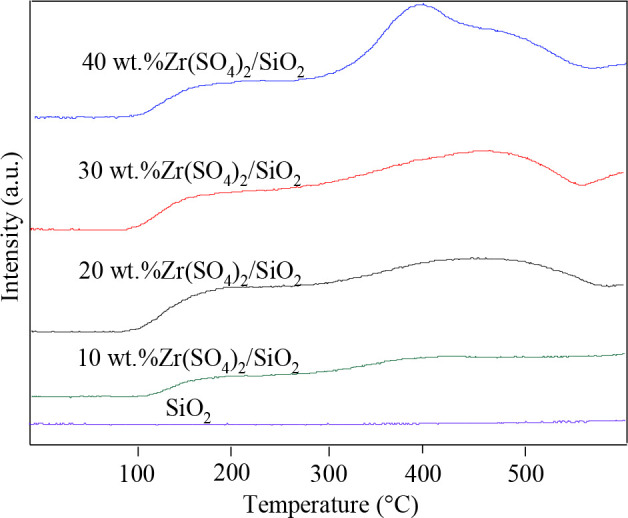
NH_3_-TPD profiles of support and catalysts with different Zr(SO_4_)_2_ loading.

The acidity of the catalysts was measured by NH_3_-TPD analysis and the results are presented in Table 4. The results show that the total acidity is proportional to Zr(SO_4_)_2_ loading. The acidity of the support SiO_2_ and catalysts with different Zr(SO_4_)_2_ loading was studied by NH_3_-TPD, and the results were shown in Figure 6. The desorption peak of the support SiO_2_ doesn^’^t appear, indicating that the prepared SiO_2 _is not acidic. The NH_3_-TPD profiles of the catalysts show desorption peaks at around 200 °C and 400 °C, and the peak area increases with the increase of Zr(SO_4_)_2_ loading, demonstrating that the acid amount increases with the increasing of Zr(SO_4_)_2_ loading. It is generally believed that desorption peaks during 150–200 °C originate from weak acid site, and the peaks above 400 °C come from strong acid sites. The prepared catalysts have both weak acid sites and strong acid sites. On the one hand, Zr^4+^ with a severe electron deficient state forms a strong coordinate bond with the oxygen atom in the combined water. On the other hand, the oxygen atom in the sulfate has a hydrogen bond with the bound water. Under the action of these two aspects, the hydrogen in the water was severely delocalized, thereby forming a strong Brønsted acid center.

**Table 4:** Catalytic performance of various catalysts for the conversions of WSP to EL.

Catalysts	Amount of acid sites (mmol×g–1)	Reaction conditions			YEL (%)	TOF(s–1)
		Temperature (°C)	Time(min)	Catalyst dosage (wt.%)		
10 wt.% Zr(SO4)2/SiO2	3.187	190	50	24	10.90	3.3 × 10–4
20 wt.% Zr(SO4)2/SiO2	4.286	190	40	20	16.37	5.5 × 10–4
30 wt.% Zr(SO4)2/SiO2	5.264	190	50	20	17.14	3.8 × 10–4
40 wt.% Zr(SO4)2/SiO2	5.487	190	50	24	15.03	2.6 × 10–4
1st-30 wt.% Zr(SO4)2/SiO2	4.052	190	50	20	15.59	4.5 × 10–4
2nd-30 wt.% Zr(SO4)2/SiO2	3.194	190	50	20	10.32	3.7 × 10–4
3rd-30 wt.% Zr(SO4)2/SiO2	3.012	190	50	20	9.21	3.5 × 10–4

### 3.2. Preparation of EL from WSP over Zr(SO4)2/SiO2

**Figure 7:**
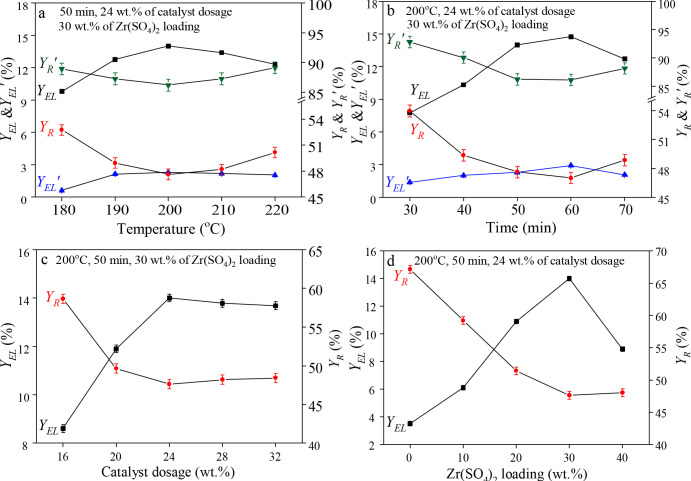
Effect of temperature, time, catalyst dosage, and Zr(SO_4_)_2_ loading on Y_EL_ and Y_R_.

The effects of temperature, time, catalyst dosage and Zr(SO_4_)_2_ loading on Y_EL_ and Y_R_ are shown in Figure 7. From Figure 7a, Y_EL_ increased firstly and then decreased at the temperature range of 180 to 220 °C, and reached the maximum of 14.01% at 200 °C. At this temperature, Y_EL_’ was 2.29%. The appropriate temperature helps break glycosidic bond in the cellulose to form intermediate products, which are subsequently converted to EL. However, when the reaction temperature exceeded 200 °C, the intermediate 5-ethoxymethyl furfural (5-EMF) decomposed easily, resulting in the decreasing of Y_EL_. During the whole reaction process, Y_R_ decreased firstly and then increased. In Figure 7b, Y_E L _raised during 30 to 60 min and had a maximum yield of 14.75% at 60 min, while Y_EL_’ was 2.91%. But the Y_EL_ decreased when the reaction time exceeded 60 min, which may be related to the polymerization of the intermediates. As Figure 7c exhibits, with the increase of catalyst dosage from 16 wt.% (16 wt.% of WSP) to 24 wt.%, Y_EL_ increased to the maximum of 14.01%, while Y_EL _decrease with the increasing of catalyst dosage from 24 wt.% to 32 wt.%. Figure 7d depicts the effect of Zr(SO_4_)_2_ loading on product yield. When Zr(SO_4_)_2_ loading increased from 0 to 30 wt.%, it was observed that Y_EL_ increased sharply from 3.53% to 14.01%, and reached the maximum of 14.01% under 30 wt.% of Zr(SO_4_)_2_ loading. More loading of Zr(SO_4_)_2_ can increase the number of acid sites, which promotes the formation of EL. However, when the loading of Zr(SO_4_)_2_ exceeds a certain value, too much acid sites can lead to an increase of side reactions, which hinders further increase of Y_EL_.

The factors affecting Y_EL_ include reaction temperature, reaction time, catalyst dosage, and Zr(SO_4_)_2 _loading. A three-level-and-four-factor orthogonal test listed in Table 5 was designed to optimize the conditions. As a result, Y_EL_ reaches 17.14% under optimum conditions (i.e. 190 °C, 50 min, 20 wt.% of catalyst dosage and 30 wt.% of Zr(SO_4_)_2_ loading). Among them, the effect of reaction temperature on Y_EL_ is the most notable. This Y_EL_ has almost unchanged compared with Y_EL _(17.91%) obtained by Chang et al. [50] using H_2_SO_4_ to catalyze wheat stalk under optimum conditions. However, our study has solved the problems that the inorganic liquid acids have serious drawbacks in the aspects of separation and recycling, as well as equipment corrosion, which makes the catalyst Zr(SO_4_)_2_/SiO_2_ a good process practicability. Moreover, WSP reaches a high conversion rate (1−Y_R_, %) under this condition, which provides a reference for the related work of catalytic conversion of wheat stalk.

**Table 5:** Orthogonal test conditions and results a.

Run	Temperature (°C)	Time(min)	Catalyst dosage(wt.%)	Zr(SO4)2 loading(wt.%)	YEL(%)
1	190	40	20	20	16.37
2	200	40	24	30	14.21
3	210	40	28	40	9.84
4	190	50	24	40	15.03
5	200	50	28	20	15.68
6	210	50	20	30	12.78
7	190	60	28	30	15.02
8	200	60	20	40	11.94
9	210	60	24	20	8.71
K1	46.42	40.42	41.09	40.76	
K2	41.83	43.49	37.95	42.01	
K3	31.33	35.67	40.54	36.81	
R	15.09	7.82	3.14	5.20	

aKi = summation of the test value of the same level, and R = (1/3Ki)max − (1/3Ki)min.

Table 4 shows the activity of various catalysts for the conversions of WSP to EL based on TOF and Y_EL_. It is obvious that 20 wt.% Zr(SO_4_)_2_/SiO_2 _shows the maximal TOF of 5.5×10^–4^ s^-1^ when reaction temperature, time, and catalyst dosage are 190 °C, 40 min, and 20 wt.%, respectively. However, 30 wt.% Zr(SO_4_)_2_/SiO_2 _presents the maximal Y_EL_ of 17.14% when reaction temperature, time, and catalyst dosage are 190 °C, 50 min, and 20 wt.%, respectively. Since TOF evaluates the catalyst activity by the amount of products generated at the unit acid site in the catalyst per unit time, the results of the catalyst activity based on TOF and Y_EL_ evaluation are small different.

### 3.3. Reaction kinetics of producing EL from WSP over Zr(SO4)2/SiO2

**Figure 8:**
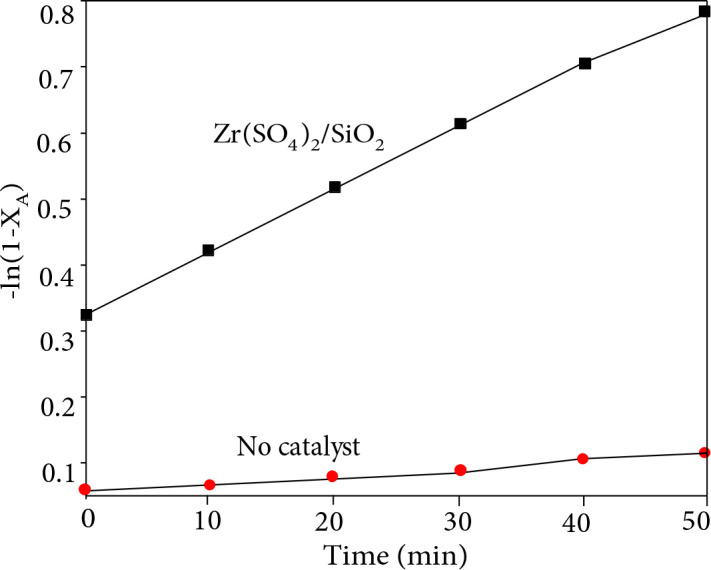
Kinetic curves of producing EL from WSP over Zr(SO_4_)_2_/SiO_2_ and no catalyst.

The reaction kinetics of producing EL from WSP over Zr(SO_4_)_2_/SiO_2_ was investigated by examining the relationship between WSP conversion (x, %) at 190 °C and reaction time. As Figure 8 shows, neglecting the impact of temperatures and catalyst deactivation on the reaction order, good linear relations between ln(1−x)^−1^ and the reaction time t suggest that the reaction of producing EL from WSP over Zr(SO_4_)_2_/SiO_2_ is a close first-order. The relation between x and t can be expressed as: t = k^−1^ln(1 −x)^−1^, where k and x are denoted as the rate constant and WSP conversion, respectively. Calculated from the slopes of ln(1−x)^−1^ versus t, rate constants were 0.075 h^–1^ and 0.019 h^–1 ^when Zr(SO_4_)_2_/SiO_2_ was used as the catalyst and without catalyst, indicating that the catalyst Zr(SO_4_)_2_/SiO_2_ has better catalytic performance for the conversion of WSP to EL.

**Figure 9:**
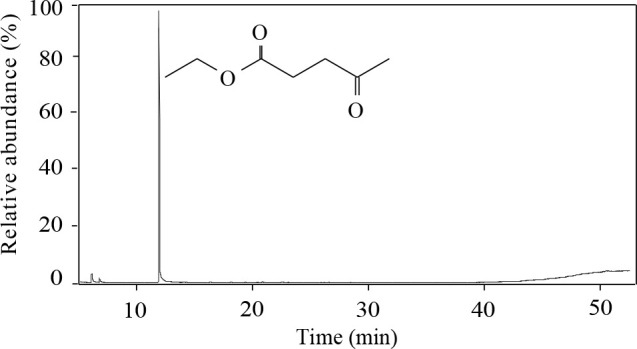
TIC of sample F_2_.

The total ion chromatogram (TIC) of sample F_2_ from the separation and purification of EL solution is shown in Figure 9. The result verifies that the main component of sample F_2_ is EL and its relative content is up to 91.73%. Further, HPLC was used to detect the composition of sample F_2_. As shown in Figure SI2, the relative content of EL reaches 90.35%, indicating that the separation method is effective.

### 3.4. Reaction pathway

**Figure 10:**
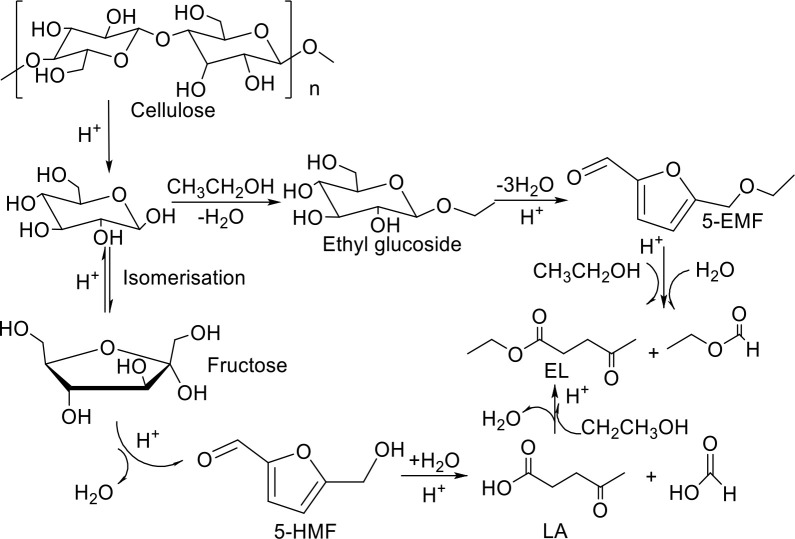
Reaction pathway in the production of EL.

Combined with the experimental results and references [51,52], the reaction pathway was speculated and shown in Figure 10. Under the attack of H^+^ from the catalyst, the glycosidic bond of cellulose in WSP breaks to afford glucose, and the conversion of glucose to EL may be through two ways. The first way is that glucose reacts with ethanol under the action of H^+^, and one molecule of water is removed to form ethyl glucoside. Ethyl glucoside is dehydrated under acidic condition to produce 5-EMF, and 5-EMF reacts with one molecule of water and one molecule of ethanol under acid conditions to afford EL. Another possible way is that glucose is firstly isomerized to fructose under acidic condition, and fructose is dehydrated under acidic condition to form 5-hydroxymethyl furfural (5-HMF). 5-HMF is hydrolyzed to form LA, and LA is esterified with ethanol to generate EL.

### 3.5. Catalyst reusability

Long-term stability of heterogeneous catalyst is an extremely important characteristic to reduce production cost in practical use. Reusability of 30 wt.% Zr(SO_4_)_2_/SiO_2_ was studied to ascertain its durability and economic viability by the method of Peng et al. [53] with slight modifications for calcination conditions (400 °C, 1.5 h), and shown in Table 4. The catalysts were recovered by calcination to remove WSP. The recovered catalysts of 30 wt.% Zr(SO_4_)_2_/SiO_2_ were denoted as 1st-30 wt.% Zr(SO_4_)_2_/SiO_2, _2nd-30 wt.% Zr(SO_4_)_2_/SiO_2,_ and 3rd-30 wt.% Zr(SO_4_)_2_/SiO_2_. After three cycles, the recovery yield of 30 wt.% Zr(SO_4_)_2_/SiO_2_ was about 92.26%, the acid sites number of 30 wt.% Zr(SO_4_)_2_/SiO_2 _decreased from 5.264 mmol×g^–1^ to 3.012 mmol×g^–1^, Y_EL _was reduced from 17.14% to 9.21%, which may be attributed to the leaching of active phase Zr(SO_4_)_2_/SiO_2_. From Figure SI3 and Table SI2, the relative content of sulfur dropped from 3.65 wt.% to 2.54 wt.% in the first run compared with the fresh 30 wt.% Zr(SO_4_)_2_/SiO_2 _catalyst. In the subsequent two cycles, the thermally regenerated catalyst was maintained at about 1.40 wt.% of sulfur relative content. In summary, the SO_4_^2–^ was partially leached from Zr(SO_4_)_2_/SiO_2 _catalyst after the first operation. In the following two cycles, the thermally regenerated catalyst was found to remain active with almost unchanged Y_EL_ and relative element content, indicating a good stability.

## 4. Conclusion

Zr(SO_4_)_2_/SiO_2_ was prepared by impregnating Zr(SO_4_)_2_ onto mesoporous silica. According to multiple characterizations, Zr(SO_4_)_2_ is successfully attached to the prepared support SiO_2 _and the acidity increases with the increasing of Zr(SO_4_)_2_ loading. Zr(SO_4_)_2_/SiO_2_ exhibits high catalytic activity for the conversion of WSP to EL. Based on catalytic performance and reaction kinetics of Zr(SO_4_)_2_/SiO_2 _research, Zr(SO_4_)_2_/SiO_2_ can effectively catalyze the conversion of WSP to EL. A comparative study showed that 20 wt.% Zr(SO_4_)_2_/SiO_2_ exhibits higher catalytic activity than other catalysts with different Zr(SO_4_)_2_ loadings. Y_EL _reaches maximum value of 17.14% when reaction temperature, reaction time, catalyst dosage, and Zr(SO_4_)_2 _loading are 190 °C, 50 min, 20 wt.% and 30 wt.%, respectively. The relative content of EL is more than 90% after three steps of distillation. This study provides an efficient way to prepare valuable chemical EL by catalytic conversion of WSP. Furthermore, high conversion of wheat stalk is obtained under this condition, which provides a reference for catalytic conversion and efficient utilization of wheat stalk.
